# Amyloid-beta aggregation implicates multiple pathways in Alzheimer’s disease: Understanding the mechanisms

**DOI:** 10.3389/fnins.2023.1081938

**Published:** 2023-04-11

**Authors:** Musa O. Iliyasu, Sunday A. Musa, Sunday B. Oladele, Abdullahi I. Iliya

**Affiliations:** ^1^Department of Anatomy, Kogi State University, Anyigba, Nigeria; ^2^Department of Human Anatomy, Ahmadu Bello University, Zaria, Nigeria; ^3^Department of Veterinary Pathology, Ahmadu Bello University, Zaria, Nigeria; ^4^Department of Human Anatomy, Federal University Dutse, Dutse, Nigeria

**Keywords:** Alzheimer’s disease, amyloid-beta, tau protein, oxidative stress, neuroinflammation, acetylcholine, mechanisms

## Abstract

Alzheimer’s disease (AD) is a progressive neurodegenerative condition characterized by tau pathology and accumulations of neurofibrillary tangles (NFTs) along with amyloid-beta (Aβ). It has been associated with neuronal damage, synaptic dysfunction, and cognitive deficits. The current review explained the molecular mechanisms behind the implications of Aβ aggregation in AD *via* multiple events. Beta (β) and gamma (γ) secretases hydrolyzed amyloid precursor protein (APP) to produce Aβ, which then clumps together to form Aβ fibrils. The fibrils increase oxidative stress, inflammatory cascade, and caspase activation to cause hyperphosphorylation of tau protein into neurofibrillary tangles (NFTs), which ultimately lead to neuronal damage. Acetylcholine (Ach) degradation is accelerated by upstream regulation of the acetylcholinesterase (AChE) enzyme, which leads to a deficiency in neurotransmitters and cognitive impairment. There are presently no efficient or disease-modifying medications for AD. It is necessary to advance AD research to suggest novel compounds for treatment and prevention. Prospectively, it might be reasonable to conduct clinical trials with unclean medicines that have a range of effects, including anti-amyloid and anti-tau, neurotransmitter modulation, anti-neuroinflammatory, neuroprotective, and cognitive enhancement.

## Introduction

The deposition of aggregated amyloid-beta (Aβ) peptide is the hallmark of Alzheimer’s disease (AD), a neurodegenerative disorder that progresses over time (Riyaz Basha et al., 2005). Due to changes in the brain and the formation of plaques and tangles, it has been linked to neuronal damage and death (Kehoe et al., 2009). AD is the most prevalent type of dementia, accounting for 60% to 70% of dementia cases among older people (Burns and Iliffe, 2009; Zhang et al., 2018).

AD symptoms include short-term memory loss, as well as a progressive decline in the patient’s capacity for thought, judgment, problem-solving, communication, and self-care (Mount and Downton, 2006; Prasansuklab and Tencomnao, 2013). The daily life of an AD patient is also impacted by symptoms including confusion, impatience, aggression, mood swings, personality and behavior changes, issues with attention and spatial orientation, and loss of long-term memory (Prasansuklab and Tencomnao, 2013).

The fifth-leading cause of death in persons over 65 years is AD (Winston, 2020). Over 26.6 million individuals worldwide suffer from it, and its prevalence is significantly increasing yearly (Prince et al., 2014; Olajide and Sarker, 2020). More than 106 million AD patients are anticipated to exist worldwide by 2050. The disease will affect 1 in 85 people, according to estimates (Brookmeyer et al., 2007), as the population ages and environmental factors take effect (Prince et al., 2014).

AD is a leading cause of disability and life reliance among elderly adults worldwide (Nichols et al., 2019), and has a profound influence on individuals, their families, and societies at large (Winston, 2020). The estimated cost of dementia in 2015 was $818 billion, or 1.1% of the GDP (Youssef et al., 2019). In 2020, it was anticipated that treating AD would cost $305 billion in total, and as the population ages, the amount is expected to rise to more than $1 trillion (Winston, 2020). The expense of dementia globally is predicted to reach $2 trillion by 2030 (Wimo et al., 2017). There are currently no effective or disease-modifying medications for AD (Fu et al., 2019). Many of the clinical trials failed in recent years, however, quite a number of the trials are under evaluation. It is essential to advance AD research to suggest new compounds for treatment and prevention. The objective of the current review is to describe the mechanisms behind the implications of Aβ aggregation in AD using multiple pathways. The literature data published between the years 1993 and 2020 were collected using PubMed and Scopus.

## Amyloid-beta

The intracellular cleavage of the amyloid precursor protein (APP) by the proteolytic enzymes beta-(β-) secretase and gamma-(γ-) secretase produces the short peptide known as Aβ, which has 40–42 amino acids (Prasansuklab and Tencomnao, 2013). The APP is localized at neuronal synapses and is abundantly expressed in the brain (Thinakaran and Koo, 2008; O’brien and Wong, 2011). It has been linked to synaptic plasticity, cell–cell or cell-matrix interactions, neuroprotection, and regulation of neuronal cell development (Storey and Cappai, 1999).

However, aggregation of Aβ, produced from the cleavage of the amyloidogenic pathway causes neurotoxicity. Most of the body’s cells, including vascular endothelial cells, thyroid epithelial cells, and neuronal and nonneuronal cultured cells, produce Aβ monomers (Schmitt et al., 1995; Fukumoto et al., 1999; Hayes et al., 2002; Kitazume et al., 2010). Although compared to other cell types, neuronal cells appear to produce more Aβ (Fukumoto et al., 1999), demonstrating the possibility that the Aβ-peptide is crucial for maintaining proper CNS physiology. According to the increased long-term potentiation (LTP) mediated by Aβ40, there is a theory that Aβ may play a crucial role in synaptic structural-functional plasticity that underlies learning and memory (Koudinov and Koudinova, 2005).

### The amyloid hypothesis

According to the amyloid hypothesis, which explains why synaptic dysfunction and neurodegeneration are brought on by the aggregation of the Aβ-peptide (Van Dyck, 2018). The main contributing factor to AD is errors in the mechanisms directing Aβ formation, accumulation, or elimination. Aβ aggregation stages impair cell-to-cell communication and stimulate the immune system, which then causes inflammation and eventually kills brain cells.

### Formation of amyloid-beta

**Figure 1:**
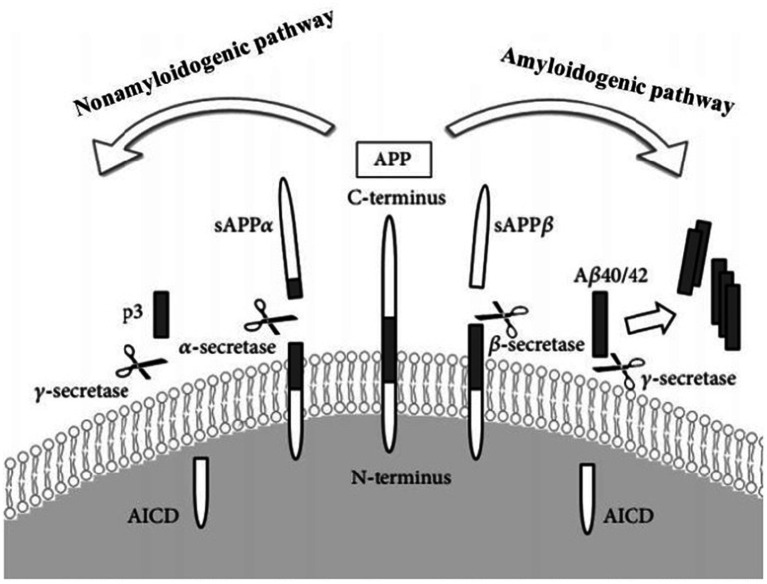
Beta-amyloid formation from the proteolytic digestion of the APP. AICD: APP intracellular C-terminal domain (Thinakaran and Koo, 2008).

The APP is processed in two distinct pathways as shown in Figure 1. Nonamyloidogenic pathway: The α-secretase enzyme first cleaves APP within the Aβ domain, and then γ-secretase cleaves at the C-terminus. Amyloidogenic pathway: Instead of α-secretase, β-secretase (BACE1) cleaves APP first at the N-terminus of the Aβ domain, and γ-secretase then cleaves it at the C-terminus. The Aβ amylogenic peptides are produced by this chain of events, which then assemble into oligomers to create extracellular neurotoxic plaques in the brain. A similar APP intracellular C-terminal domain (AICD) is released from both pathways (Thinakaran and Koo, 2008). When compared to other fragments, Aβ is chemically “stickier” than those formed by APP proteolytic processes. Small clusters (oligomers) are formed by the fragments initially, followed by chains of clusters (fibrils), and finally “mats” of fibrils (beta-sheets). The final stage is the forming of plaques which contain clusters of beta-sheets and other chemicals (Jung et al., 2010). The amyloid cascade hypothesis (ACH) explains AD pathogenesis from the outcome of two significant facts: (i) Identification of Aβ as a key component of senile plaques (SPs). (ii) Mutations of APP genes and the presenilin 1 and 2 genes (PSEN1 and PSEN2) which are typically detected at the early stage of AD. As a result, it is believed that the emergence of Aβ within SPs is caused by these mutations, which also cause dementia and neuronal cell death (Reitz, 2012).

### Formation of neurofibrillary tangles

The aggregation of Aβ causes the formation of neurofibrillary tangles (NFTs) from hyperphosphorylation of tau and its accumulation into tangles is another pathological cause of AD (McGleenon et al., 2009). In normal conditions, tau supports neuronal structures and functions in the brain (Kolarova et al., 2012). However, under pathological circumstances, tau became excessively hyperphosphorylated and aggregated into fibrils known as neurofibrillary tangles. The accumulation of abnormal tau and tangles in neurons leads to neurotoxicity and neuronal degeneration (Gómez-Isla et al., 1997). In addition to the formation of NFTs, Tau phosphorylation impairs tau’s ability to bind microtubules, which impacts neuronal activities such as axonal transport and mitochondrial respiration (Ittner and Götz, 2011). Microtubule depolymerization, self-aggregation, and detachment caused by tau hyperphosphorylation result in neuronal cell death (Suganthy et al., 2016).

## Mechanisms of Alzheimer disease

**Figure 2:**
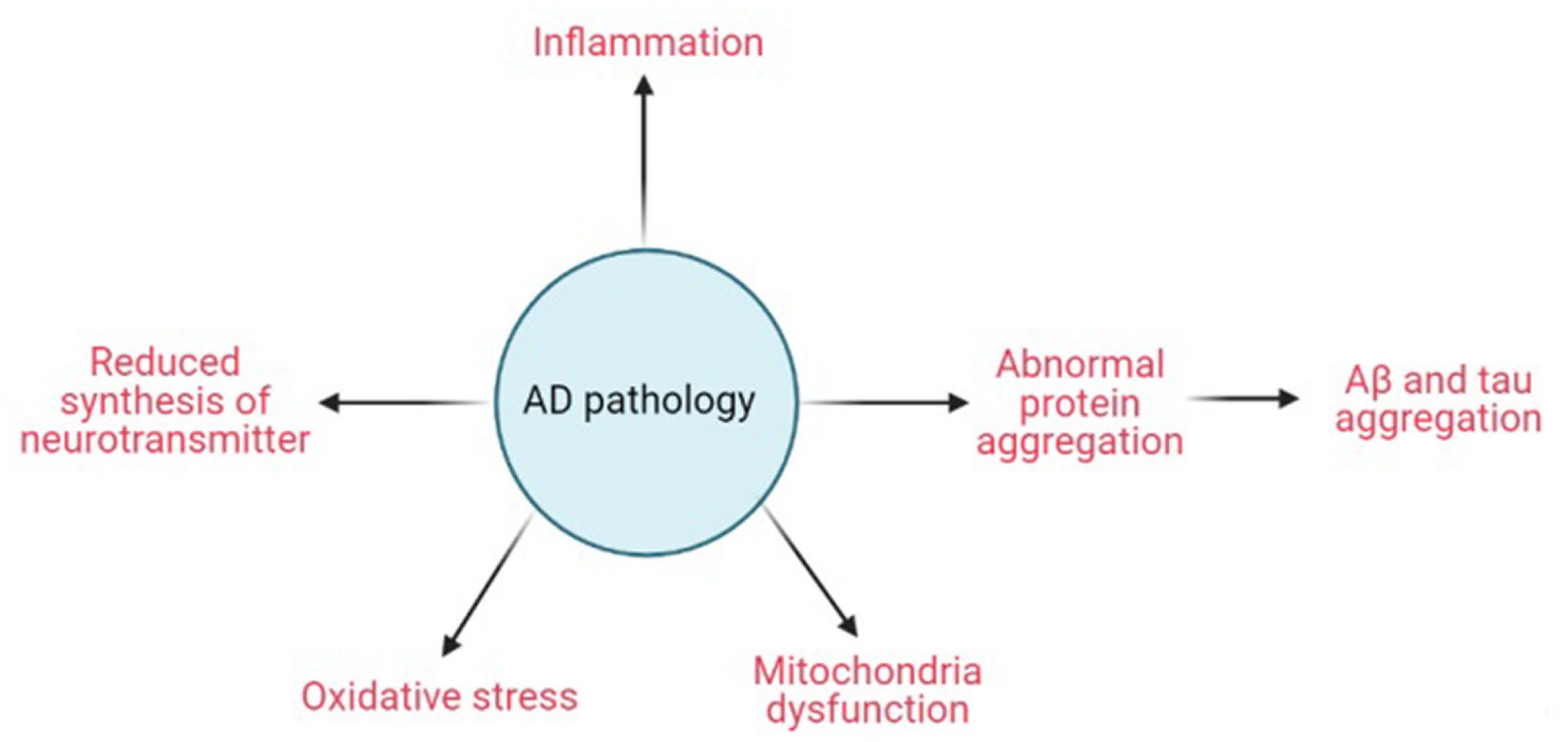
Schematic diagram of AD pathology. Created with BioRender.com.

AD pathogenesis starts from the deposition of Aβ which trigger SPs formation, followed by the death of neurons due to NFTs formation (Armstrong, 2011). Neurotoxic mechanisms in the pathology of AD include aberrant protein aggregation, dysfunction of mitochondrial, decreased neurotransmitter production, inflammation, and oxidative stress (Figure 2). However, the buildup of Aβ and the aggregation of tau are the two most prevalent etiologic models of Alzheimer’s pathogenesis (Bloom, 2014).

**Figure 3:**
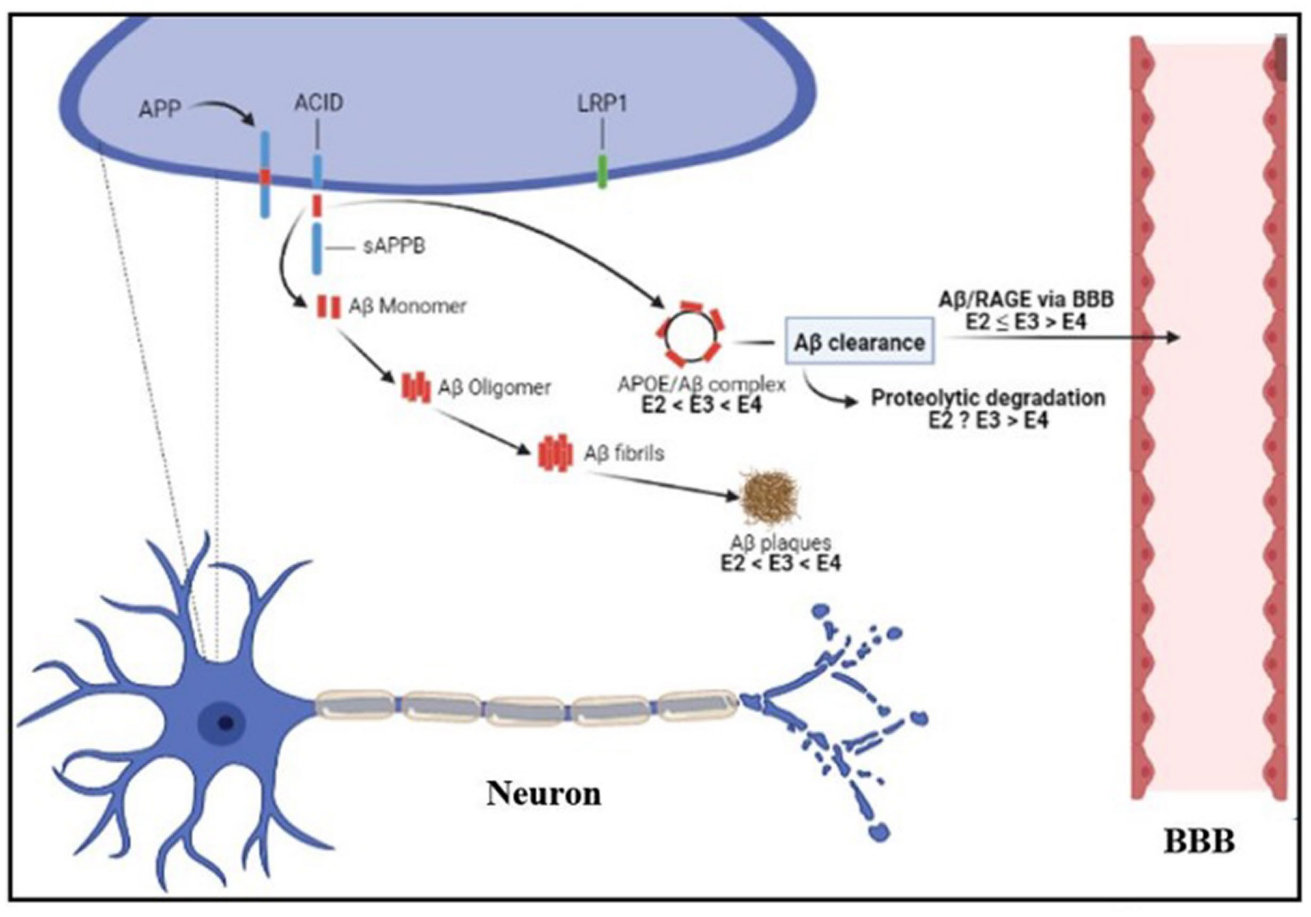
Diagrammatic representation of the regulatory systems for Aβ in an AD patient’s brain. Aβ, amyloid-beta; BBB, Blood–brain barrier; RAGE, Receptor for advanced glycation end products; AICD, APP intracellular C-terminal domain; APP, Amyloid precursor protein; ApoE, apolipoprotein E; E4, ApoE; E3, ApoE3; E2, ApoE2. Created with BioRender.com.

The neuropathological events in AD patients are the result of the toxicity of amyloid oligomers and fibrils, which are from the aggregated forms of Aβ. The bodies regulate the amyloid level *via* a variety of methods as Aβ accumulates. The concentration of Aβ-peptide is controlled in healthy brain tissue by its production from APP; the influx across the blood–brain barrier (BBB) *via* its interaction with the receptor for advanced glycation end products (RAGE; Deane et al., 2003, 2009); and its clearance *via* the low-density lipoprotein receptor-related protein-1 (LRP1) from the brain and enzymatic breakdown in the brain (Selkoe, 2001; Deane et al., 2003, 2009). Additionally, the levels of Aβ affect how RAGE is expressed. RAGE is upregulated when there is excessive Aβ synthesis, and this leads to neurotoxicity (Prasansuklab and Tencomnao, 2013) as shown in Figure 3. Thus, impairments in these regulatory processes may cause excessive Aβ-peptide to build up and deposit in the brains of AD patients. By binding to Aβ_12-28_ residues, apolipoprotein E (ApoE) regulates Aβ’s accumulation and lessens its clearance (Prasansuklab and Tencomnao, 2013; Zhang et al., 2018) from the brain (Sagare et al., 2007). Three isoforms of ApoE such as ApoE4 (E4), ApoE3 (E3), and ApoE2 (E2; Liu et al., 2013), regulate cholesterol levels in various ways to influence γ-secretase activity and Aβ synthesis (Osenkowski et al., 2008). According to Bales et al. (2009) and Castellano et al. (2011), the brain Aβ levels and amyloid plaque loading rely on the ApoE isoforms, demonstrating the modulatory involvement of ApoE in Aβ metabolism, aggregation, and deposition (Liu et al., 2013). The differential lipidation status exhibited by ApoE isoforms affects Aβ clearance. The ApoE particles may seize Aβ and stimulate cellular uptake and degradation of ApoE-Aβ complexes (Kim et al., 2009). Aβ clearance at the blood–brain barrier is inhibited by ApoE in an isoform-dependent manner (E4 > E3 and E2). According to studies, E4 is less effective than E3 and E2 at mediating the clearance of Aβ (Deane et al., 2008; Jiang et al., 2008).

### Effects of metal ions on Aβ and tau aggregation

**Figure 4:**
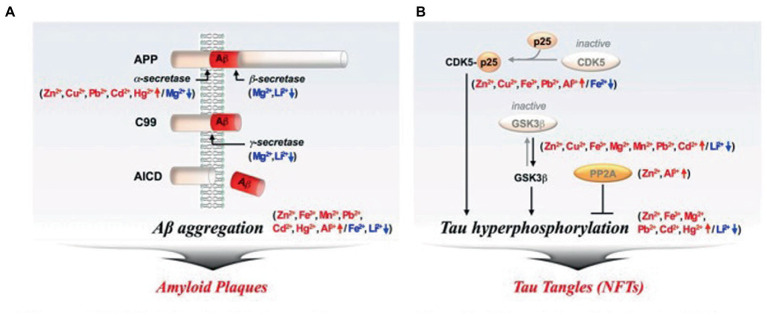
Metal ions’ effects on the aggregation of Aβ and tau. **(A)** Amyloid Plaques, **(B)** Tau tangle. Aβ, amyloid-beta; CDK5, Cyclin-dependent kinase; GSK-3β, Glycogen synthase kinase-3beta; NFTs, Neurofibrillary tangles; PP2A, protein phosphatase 2A (O’brien and Wong, 2011).

Strong neurotoxic candidates that alter Aβ and tau aggregation include metal dyshomeostasis (Figure 4). Metal ions’ effects on the aggregation of Aβ and tau have been elucidated. Metals like Zn^2+^, Cu^2+^, Fe^3+^, Mn^2+^, Pb^2+^, Cd^2+^, Hg^2+^, and Al^3+^ stimulate amyloidogenic pathways and Aβ aggregation. [red arrow] (O’brien and Wong, 2011). The neurotoxic Aβ-peptide produced by the cleavage of the APP by β- and γ-secretase is secreted into the extracellular space where it spontaneously changes into amyloid plaques. On the other hand, as seen in Figure 4A, the presence of Mg^2+^, Fe^2+^, and Li^2+^ inhibits the production of Aβ [blue arrow] (O’brien and Wong, 2011).

Tau hyperphosphorylation and aggregation are promoted by metal ions like Zn^2+^, Cu^2+^, Fe^3+^, Mg^2+^, Mn^2+^, Pb^2+^, Cd^2+^, Hg^2+^, and Al^3+^ [red arrow]. Numerous kinases, including glycogen synthase kinase-3 beta (GSK-3β; Rankin et al., 2007), cyclin-dependent kinase 5 (CDK-5), and others, strongly phosphorylate tau (Kimura et al., 2014). If protein phosphatase 2A (PP2A) is not activated, the hyperphosphorylation of tau may persist (Goedert, 1993). Tau that has been hyperphosphorylated forms NFTs. As depicted in Figure 4B, metal ions like Fe^2+^, and Li^2+^, however, lessen tau hyperphosphorylation [blue arrow].

### Induction of oxidative stress by Aβ in AD

**Figure 5:**
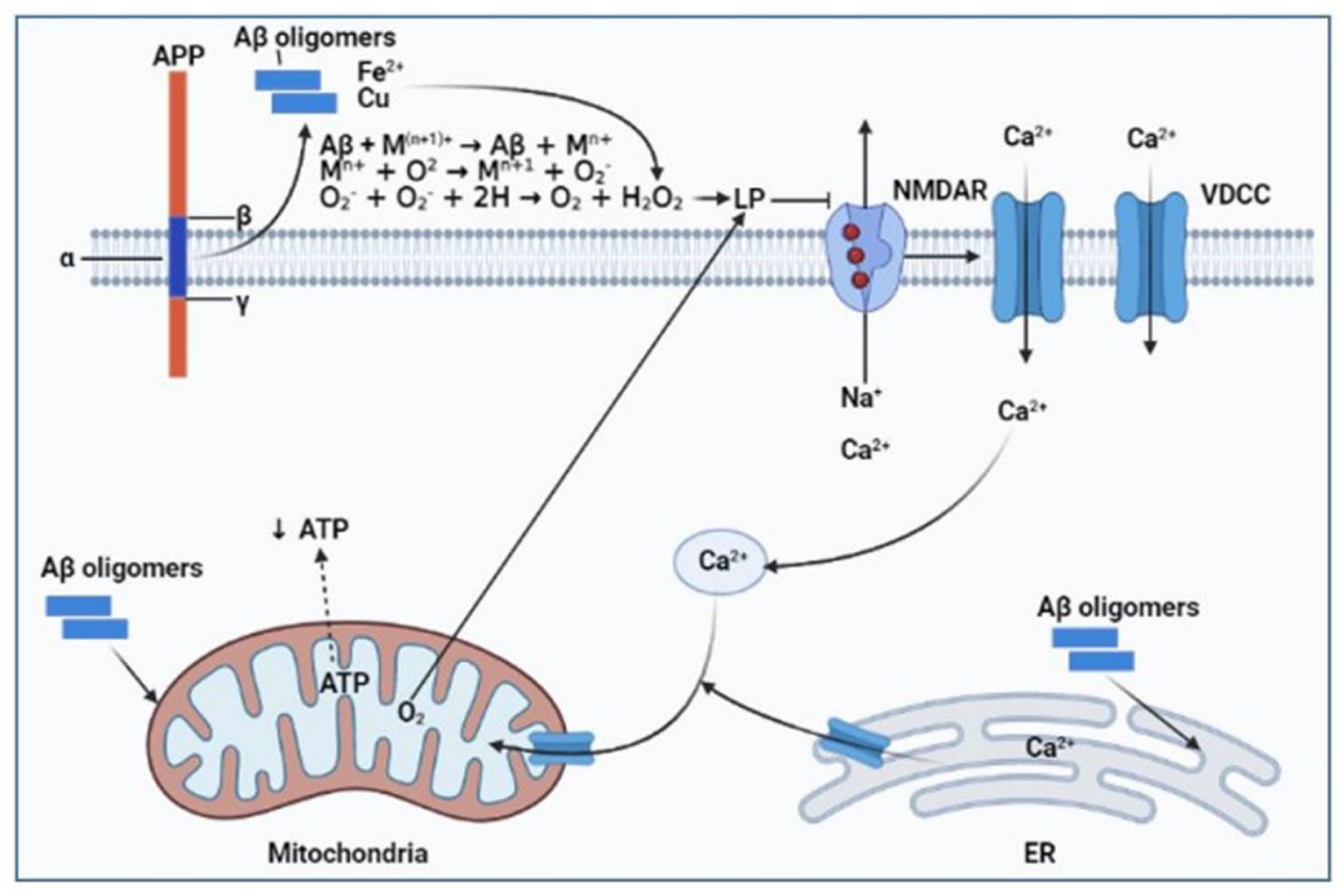
Diagram showing how Aβ and metal ions combine to cause oxidative stress in AD. Aβ, amyloid-beta; LP, Lipid peroxidation; NMDAR, N-methyl-D-aspartate receptor; VDCC, Voltage-dependent calcium channel; ER, endoplasmic reticulum; APP, Amyloid precursor protein; ATP, Adenosine triphosphate. Created with BioRender.com.

The polymeric forms of Aβ cause alterations in biochemical components and brain cell activities that lead to neuropathology associated with AD symptoms. According to reports, one of the earliest clinical manifestations of AD is increased oxidative stress. Hydrogen peroxide (H_2_O_2_) created due to the reduction of metal ions by Aβ-peptides served as a mediator of the oxidative stress as shown in Figure 5 (Huang et al., 1999; Atwood et al., 2003). Aβ-peptides act as powerful oxidation catalysts and can capture transition metal ions like Cu, Fe, and Zn (Miura et al., 2000). In addition, it was shown that Aβ was toxic to neuronal cultures, and Cu^2+^ ions made it more toxic (Cuajungco et al., 2000). Reactive oxygen species can be produced by the Aβ/Cu(Fe) complexes as a toxin mediator (Huang et al., 1999). Furthermore, AD brains have an extracellular and intracellular accumulation of metal ions with high concentrations of Aβ plaques (Lovell et al., 1998; Religa et al., 2006), which produced free radicals. Because of lipid peroxidation and oxidative protein modification, several biomolecules in the AD brain experience conformational and structural changes that impair their ability to function, which, in turn, affects a variety of cellular processes (Qi et al., 2005). By upregulating the expression of the BACE1 gene, increasing oxidative stress enhances APP processing and ultimately increases Aβ generation (Tong et al., 2005; Coma et al., 2008; Quiroz-Baez et al., 2009). This causes oxidative stress and endoplasmic reticulum (ER) stress by increasing ROS and the accompanying rise in abnormal APP and phosphorylated tau. The ER function can be severely damaged by long-term ER stress, which also causes apoptotic signaling (Ogata et al., 2006; Kouroku et al., 2007). Aβ promotes Ca^2+^ release from neurons’ ER Ca^2+^ pools, increasing intracellular free Ca^2+^ (Zhou et al., 2020). Increased expression of the NR2B subunit of NMDAR causes a rise in Ca^2+^ ion concentration in extrasynaptic regions (Jusko et al., 2008), which, then increases the level of intracellular endoplasmic reticulum Ca^2+^ production (Yin et al., 1994). Studies revealed that Ca^2+^ overload could increase ER stress and facilitate mitochondrial Ca^2+^ uptake by suppressing the expression of the anti-apoptotic protein B-cell lymphoma 2 (Bcl2) and increasing the phosphorylation of extracellular regulated protein kinases (Erk) protein, which would ultimately lead to cytotoxicity and cellular apoptosis (Hajnóczky et al., 2003; Zieg et al., 2008; Zhang et al., 2018).

### Induction of neuroinflammation by Aβ in AD

**Figure 6:**
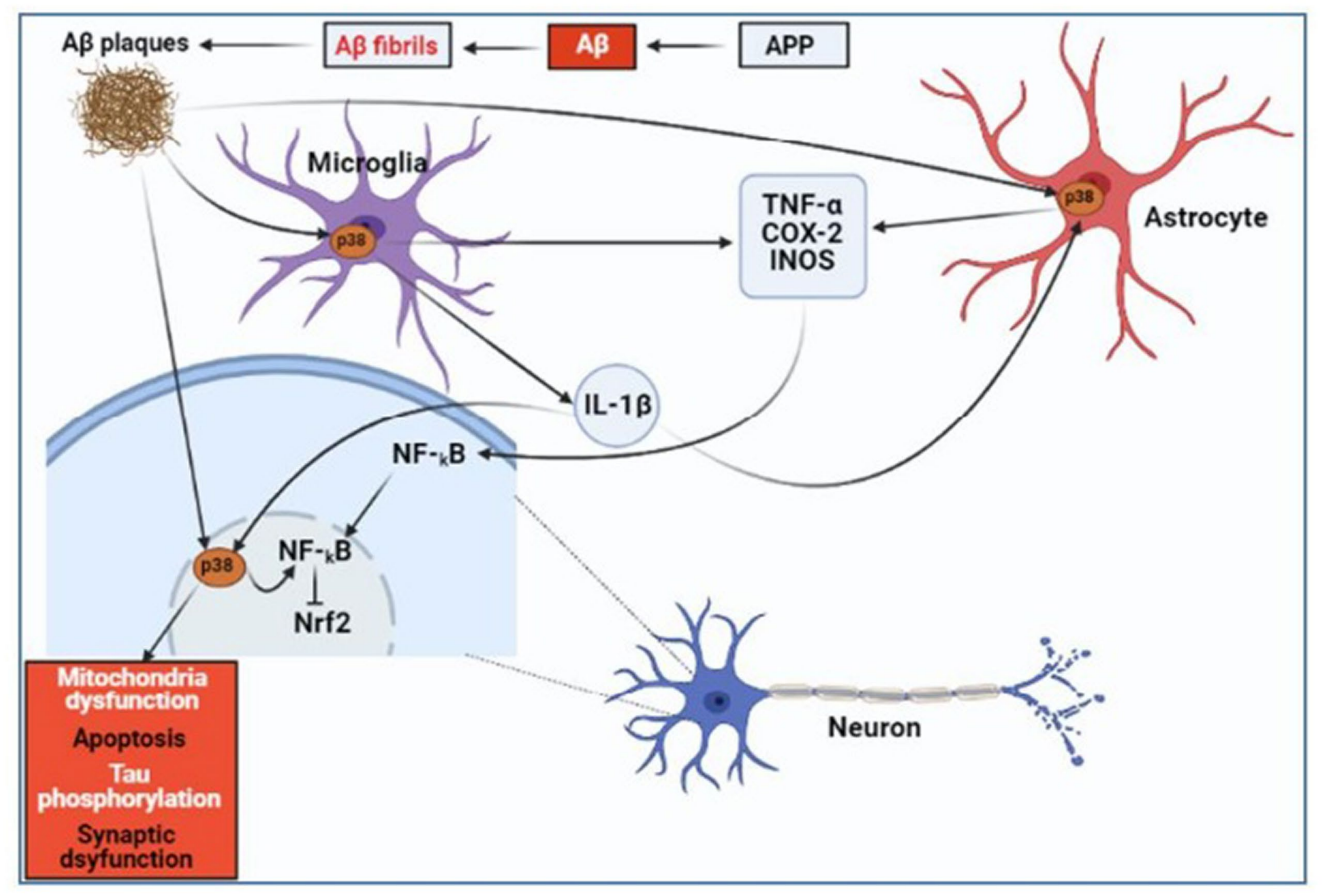
Diagrammatic representation of how Aβ causes neuroinflammation in AD. Aβ, amyloid-beta; NF-κB, Nuclear factor-κB; p38 MAPK, p38 Mitogen-activated protein kinases; Nrf2, Nuclear factor E2-related factor 2; IL-1β, Interleukin-1β; TNF-α, Tumor necrosis factor-α; COX-2, Cyclooxygenase-2; and iNOS, Inducible nitric oxide synthase (Schnöder et al., 2016; Lee and Kim, 2017). Created with BioRender.com.

The expression of pro-inflammatory cytokines was increased in response to neuropathological insults induced by Aβ and its interaction with vascular RAGE (Deane et al., 2008). Microglia enhance the clearance of Aβ, but a constant generation of Aβ causes the microglia to become chronically activated, which promotes more amyloid deposition (Hickman et al., 2018). According to Kim and Choi (2015), exposure to Aβ results in microglial activation, which, in turn, causes the generation of reactive oxygen species and neurotoxic pro-inflammatory cytokines. Tau hyperphosphorylation is a result of ROS-activating p38 mitogen-activated protein kinases (p38 MAPK; Giraldo et al., 2014). p38 MAPK has been linked to neuroinflammation and AD due to its ability to activate nuclear factor-B (NF-κB; Kheiri et al., 2018), a master regulator of neuroinflammation gene transcription in the brains of AD patients (Chen et al., 2012; Liao et al., 2016; Olajide and Sarker, 2020). But data indicate that nuclear factor E2-related factor 2 (Nrf2) is negatively regulated by NF-κB (Liu et al., 2008; Kim and Vaziri, 2010; Yu et al., 2011). Substantial evidence connects the activation of the Nrf2 protection mechanism to NF-κB-mediated inflammatory actions (Nair et al., 2008; Sandberg et al., 2014). To uphold the aforementioned finding, Rojo et al. (2010) showed that cyclooxygenase-2 (COX-2), inducible nitric oxide synthases (iNOS), IL-6, and TNF-α levels are elevated when microglia are activated in Nrf2-deficient rats. Ramsey et al. (2007) first noticed this, reporting that the hippocampus of AD patients’ brains had lower amounts of Nrf2 than normal. According to Lee and Kim (2017), through the activation of p38 MAPK, Aβ plaques cause neuronal impairments such as mitochondrial dysfunction, apoptosis, tau phosphorylation, and synaptic dysfunction; the primary cause of neuroinflammation in AD is increased microglial p38 MAPK signaling brought on by Aβ, which results in the production of pro-inflammatory mediators such interleukin-1β (IL-1β), tumor necrosis factor-α (TNF-α), cyclooxygenase-2 (COX-2), and inducible nitric oxide synthase (iNOS); the pathophysiology of the AD brain is worsened by the production of IL-1β from microglia, which increases p38 MAPK activation in neurons and astrocytes; Aβ plaques and IL-1β generated an increase in P38 MAPK activation in astrocytes. By releasing iNOS, COX-2, and TNF-α, this activation accelerates neuroinflammation (Figure 6).

### Effects of Aβ on acetylcholine in AD

**Figure 7:**
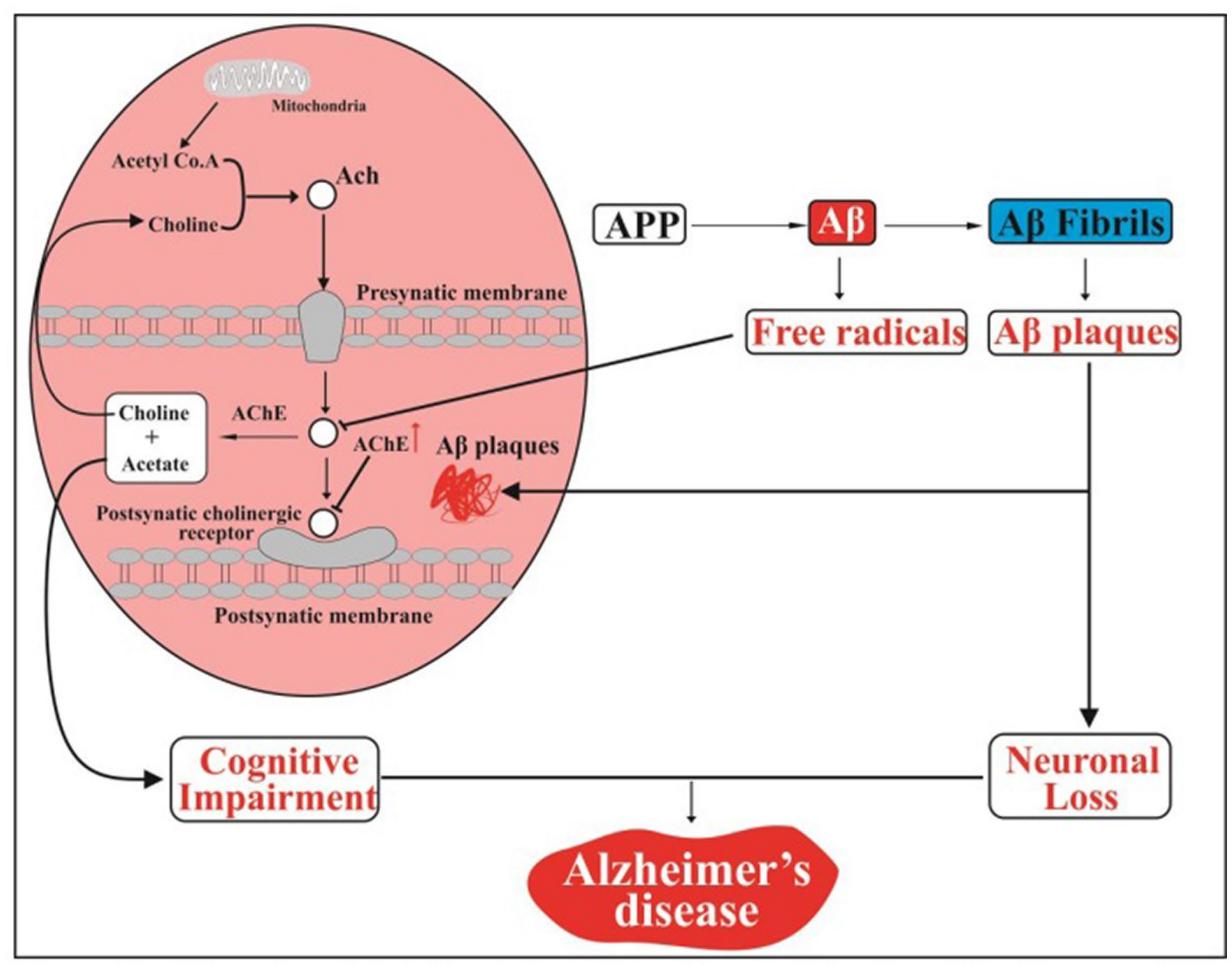
Aβ and acetylcholine interactions in an AD schematic diagram. APP, Amyloid precursor protein; Aβ, Amyloid-beta; Ach, acetylcholine; AChE, acetylcholinesterase (DeTure and Dickson, 2019). Created with BioRender.com.

As a neurotransmitter, acetylcholine (Ach) aids in the communication between nerve cells and is essential for memory and learning processes (Kihara and Shimohama, 2004; Francis, 2005). A report revealed that Alzheimer’s patients have reduced amounts of Ach in their brains (Kihara and Shimohama, 2004). Ach is decreased because oxidative stress is induced and inflammatory cytokines are produced by Aβ (Esposito et al., 2006). Free radicals produced due to amyloid peptides have been shown to lower the concentration of Ach by causing cholinergic neurons in the hippocampus to degenerate (Vinod et al., 2009). Additionally, acetylcholinesterase (AChE) activity increases and deactivates acetylcholine in synaptic clefts in the vicinity of amyloid plaques (Mordn et al., 1993; Sberna et al., 1997). According to another study, the amyloid peptide inhibits the production of acetylcholine (ACh) by causing choline to seep through cell membranes (Ehrenstein et al., 1997). Ach deficiency caused cognitive impairment and ultimately AD (Parent et al., 2013; Deture and Dickson, 2019) as shown in Figure 7.

### Current state of AD treatment

According to Yiannopoulou and Papageorgiou (2013), the formation of amyloid oligomers, which mediates the amyloid cascade, is primarily responsible for neurotoxicity. The main pathophysiologic pillars are oxidation, inflammation, excessive glutamate, and tau hyperphosphorylation. Anti-amyloid disease-modifying treatments (DMTs) have therefore concentrated on three main mechanisms of action (MOAs), including reducing the formation of Aβ_42_, reducing the burden of Aβ-plaque, and promoting Aβ clearance (Yiannopoulou and Papageorgiou, 2020). Hence, inhibiting the formation of A𝛽-peptide accumulation and tau hyperphosphorylation may be part of the treatment for AD (Mendiola-Precoma et al., 2016). Physical exercise, a healthy diet, and mental stimulation are further AD prevention strategies (Nelson and Tabet, 2015).

### Compounds used in clinical trials for the treatment of AD

Acetylcholinesterase inhibitors (AChEIs), such as rivastigmine, donepezil, and galantamine, are clinically effective in increasing the availability of acetylcholine at synapses and thereby inhibiting cognitive decline in AD (Andrieu et al., 2015; Hampel et al., 2018; Cummings et al., 2019). Nevertheless, diarrhea, nausea, and vomiting are some of the typical negative effects of AChEIs on the digestive system (Yiannopoulou and Papageorgiou, 2020). Memantine, which was approved in 2003, selectively binds to open calcium channels that are controlled by NMDA receptors, inhibiting NMDA-mediated ion flux and reducing pathologically excessive glutamate levels (Yiannopoulou and Papageorgiou, 2013; Matsunaga et al., 2015; Cummings et al., 2019). Memantine also reduces the activity of glycogen synthase kinase 3𝛽 (GSK-3𝛽), which, in turn, reduces tau phosphorylation (Prentice et al., 2015; Folch et al., 2016).

Despite extensive and expensive trials, the Food and Drug Administration (FDA) has not approved any DMTs or new medications for AD since 2003 (Anderson et al., 2017; Hukins et al., 2019). The β-secretase (BACE) inhibitors, lanabecestat (Burki, 2018), verubecestat (Egan et al., 2019), and atabecestat (Henley et al., 2019), as well as the anti-amyloid agents such as semagacestat (Doody et al., 2013), bapineuzumab (Vandenberghe et al., 2016), and solanezumab (Neurology, 2016), failed in recent phase 3 clinical trials. The acknowledged explanations for the numerous failures include inadequate understanding of the pathophysiology, inappropriate drug doses, late therapies in disease progression, and wrong therapeutic targets (Gauthier et al., 2016).

## Conclusion

The current review explained the molecular mechanisms of Aβ mediating AD *via* multiple events, including Aβ production and accumulation, tau hyperphosphorylation, metal dyshomeostasis, oxidative stress, neuroinflammation, and inhibition of acetylcholine production. There are presently no efficient or disease-modifying medications for AD. Some of the clinical trials targeting the above events failed in recent years, however, quite a number of the trials are under evaluation. It is necessary to advance AD research to suggest novel compounds for treatment and prevention. Prospectively, it might be reasonable to conduct clinical trials with unclean medicines that have a range of effects, including anti-amyloid, anti-tau, neurotransmitter modulation, anti-neuroinflammatory, neuroprotective, and cognitive enhancement.

## Author contributions

MI wrote the manuscript. SM, SO, and AI edited, approved, and concluded the manuscript. All authors contributed to the article and approved the submitted version.

## Conflict of interest

The authors declare that the research was conducted in the absence of any commercial or financial relationships that could be construed as a potential conflict of interest.

## Publisher’s note

All claims expressed in this article are solely those of the authors and do not necessarily represent those of their affiliated organizations, or those of the publisher, the editors and the reviewers. Any product that may be evaluated in this article, or claim that may be made by its manufacturer, is not guaranteed or endorsed by the publisher.

## References

[ref2] Anderson R. M. Hadjichrysanthou C. Evans S. Wong M. M. (2017). Why do so many clinical trials of therapies for Alzheimer’s disease fail? Lancet 390, 2327–2329. doi: 10.1016/S0140-6736(17)32399-1, PMID: 29185425

[ref3] Andrieu S. Coley N. Lovestone S. Aisen P. S. Vellas B. (2015). Prevention of sporadic Alzheimer’s disease: lessons learned from clinical trials and future directions. Lancet Neurol. 14, 926–944. doi: 10.1016/S1474-4422(15)00153-2, PMID: 26213339

[ref4] Armstrong R. A. (2011). The pathogenesis of alzheimer’s disease: A reevaluation of the “amyloid cascade hypothesis”. Int. J. Alzheimers Dis. 2011, 1–6. doi: 10.4061/2011/630865, PMID: 21331369 PMC3038555

[ref5] Atwood C. S. Obrenovich M. E. Liu T. Chan H. Perry G. Smith M. A.. (2003). Amyloid-β: A chameleon walking in two worlds: A review of the trophic and toxic properties of amyloid-β. Brain Res. Rev. 43, 1–16. doi: 10.1016/S0165-0173(03)00174-7, PMID: 14499458

[ref6] Bales K. R. Liu F. Wu S. Lin S. Koger D. DeLong C.. (2009). Human APOE isoform-dependent effects on brain β-amyloid levels in PDAPP transgenic mice. J. Neurosci. 29, 6771–6779. doi: 10.1523/JNEUROSCI.0887-09.2009, PMID: 19474305 PMC6665579

[ref7] Bloom G. S. (2014). Amyloid-β and tau: the trigger and bullet in Alzheimer’s disease pathogenesis. JAMA Neurol. 71, 505–508. doi: 10.1001/jamaneurol.2013.5847 24493463 PMC12908160

[ref8] Brookmeyer R. Johnson E. Ziegler-Graham K. Arrighi H. M. (2007). Forecasting the global burden of Alzheimer’s disease. Alzheimers Dement. 3, 186–191. doi: 10.1016/j.jalz.2007.04.381 19595937

[ref9] Burki T. (2018). Alzheimer’s disease research: the future of BACE inhibitors. Lancet 391:2486. doi: 10.1016/S0140-6736(18)31425-9, PMID: 29976459

[ref10] Burns A. Iliffe S. (2009). Alzheimer’s disease. BMJ 338, 467–471. doi: 10.1136/bmj.b158 19196745

[ref11] Castellano J. M. Kim J. Stewart F. R. Jiang H. DeMattos R. B. Patterson B. W.. (2011). Human apoE isoforms differentially regulate brain amyloid-β peptide clearance. Sci. Transl. Med. 3:89ra57. doi: 10.1126/scitranslmed.3002156, PMID: 21715678 PMC3192364

[ref12] Chen C. H. Zhou W. Liu S. Deng Y. Cai F. Tone M.. (2012). Increased NF-κB signalling up-regulates BACE1 expression and its therapeutic potential in Alzheimer’s disease. Int. J. Neuropsychopharmacol. 15, 77–90. doi: 10.1017/S1461145711000149, PMID: 21329555

[ref13] Coma M. Guix F. X. Ill-Raga G. Uribesalgo I. Alameda F. Valverde M. A.. (2008). Oxidative stress triggers the amyloidogenic pathway in human vascular smooth muscle cells. Neurobiol. Aging 29, 969–980. doi: 10.1016/j.neurobiolaging.2007.01.009, PMID: 17306421

[ref14] Cuajungco M. P. Goldstein L. E. Nunomura A. Smith M. A. Lim J. T. Atwood C. S.. (2000). Evidence that the β-amyloid plaques of Alzheimer’s disease represent the redox-silencing and entombment of Aβ by zinc. J. Biol. Chem. 275, 19439–19442. doi: 10.1074/jbc.C000165200, PMID: 10801774

[ref15] Cummings J. Lee G. Ritter A. Sabbagh M. Zhong K. (2019). Alzheimer’ s disease drug development pipeline: 2019. Alzheimers Dement. 5, 272–293. doi: 10.1016/j.trci.2019.05.008, PMID: 31334330 PMC6617248

[ref16] Deane R. Bell R. D. Sagare A. Zlokovic B. V. (2009). Clearance of amyloid-β peptide across the blood-brain barrier: implication for therapies in Alzheimer’s disease. CNS Neurol. Disord. Drug Targets 8, 16–30. doi: 10.2174/187152709787601867, PMID: 19275634 PMC2872930

[ref17] Deane R. du Yan S. Submamaryan R. K. LaRue B. Jovanovic S. Hogg E.. (2003). RAGE mediates amyloid-β peptide transport across the blood-brain barrier and accumulation in brain. Nat. Med. 9, 907–913. doi: 10.1038/nm890, PMID: 12808450

[ref18] Deane R. Sagare A. Hamm K. Parisi M. Lane S. Finn M. B.. (2008). apoE isoform-specific disruption of amyloid β peptide clearance from mouse brain. J. Clin. Investig. 118, 4002–4013. doi: 10.1172/JCI36663, PMID: 19033669 PMC2582453

[ref19] Deture M. A. Dickson D. W. (2019). The neuropathological diagnosis of Alzheimer’s disease. Mol. Neurodegener. 14, 32–18. doi: 10.1186/s13024-019-0333-5, PMID: 31375134 PMC6679484

[ref20] Doody R. S. Raman R. Farlow M. Iwatsubo T. Vellas B. Joffe S.. (2013). A phase 3 trial of Semagacestat for treatment of Alzheimer’s disease. N. Engl. J. Med. 369, 341–350. doi: 10.1056/nejmoa1210951, PMID: 23883379

[ref21] Egan M. F. Kost J. Tiffini Voss M. D. Yuki Mukai M. D. Paul S. Aisen M. D.. (2019). Randomized trial of Verubecestat for prodromal Alzheimer’s disease. N. Engl. J. Med. 380, 1408–1420. doi: 10.1056/NEJMoa1812840.Randomized, PMID: 30970186 PMC6776078

[ref22] Ehrenstein G. Galdzicki Z. Lange G. D. (1997). The choline-leakage hypothesis for the loss of acetylcholine in Alzheimer’s disease. Biophys. J. 73, 1276–1280. doi: 10.1016/S0006-3495(97)78160-8, PMID: 9284295 PMC1181027

[ref23] Esposito L. Raber J. Kekonius L. Yan F. Yu G. Q. Bien-Ly N.. (2006). Reduction in mitochondrial superoxide dismutase modulates Alzheimer’s disease-like pathology and accelerates the onset of behavioral changes in human amyloid precursor protein transgenic mice. J. Neurosci. 26, 5167–5179. doi: 10.1523/JNEUROSCI.0482-06.2006, PMID: 16687508 PMC6674260

[ref24] Folch J. Petrov D. Ettcheto M. Abad S. Sánchez-López E. García M. L.. (2016). Current research therapeutic strategies for Alzheimer’s disease treatment. Neural Plast. 63, 390–401. doi: 10.1016/0012-1606(78)90143-4 PMC4735913 26881137

[ref25] Francis P. T. (2005). The interplay of neurotransmitters in Alzheimer’s disease. CNS Spectr. 10, 6–9. doi: 10.1017/s1092852900014164 16273023

[ref26] Fu W. Y. Wang X. Ip N. Y. (2019). Targeting neuroinflammation as a therapeutic strategy for Alzheimer’s disease: mechanisms, drug candidates, and new opportunities. ACS Chem. Nerosci. 10, 872–879. doi: 10.1021/acschemneuro.8b00402, PMID: 30221933

[ref27] Fukumoto H. Tomita T. Matsunaga H. Ishibashi Y. Saido T. C. Iwatsubo T. (1999). Primary cultures of neuronal and non-neuronal rat brain cells secrete similar proportions of amyloid β peptides ending at Aβ40 and Aβ42. Neuroreport 10, 2965–2969. doi: 10.1097/00001756-199909290-00017, PMID: 10549806

[ref28] Gauthier S. Albert M. Fox N. Goedert M. Kivipelto M. Mestre-Ferrandiz J.. (2016). Why has therapy development for dementia failed in the last two decades? Alzheimers Dement. 12, 60–64. doi: 10.1016/j.jalz.2015.12.003, PMID: 26710325

[ref29] Giraldo E. Lloret A. Fuchsberger T. Viña J. (2014). Aβ and tau toxicities in Alzheimer’s are linked via oxidative stress-induced p38 activation: protective role of vitamin E. Redox Biol. 2, 873–877. doi: 10.1016/j.redox.2014.03.002, PMID: 25061569 PMC4099506

[ref30] Goedert M. (1993). Tau protein and the neurofibrillary pathology of Alzheimer’s disease. Trends Neurosci. 16, 460–465. doi: 10.1016/0166-2236(93)90078-Z 7507619

[ref31] Gómez-Isla T. Hollister R. West H. Mui S. Growdon J. H. Petersen R. C.. (1997). Neuronal loss correlates with but exceeds neurofibrillary tangles in Alzheimer's disease. Ann. Neurol. 41, 17–24. doi: 10.1002/ana.410410106, PMID: 9005861

[ref32] Hajnóczky G. Davies E. Madesh M. (2003). Calcium signaling and apoptosis. Biochem. Biophys. Res. Commun. 304, 445–454. doi: 10.1016/S0006-291X(03)00616-8 12729578

[ref33] Hampel H. Mesulam M. Cuello A. C. Farlow M. R. Giacobini E. Grossberg G. T.. (2018). The cholinergic system in the pathophysiology and treatment of Alzheimer’s disease. Brain 141, 1917–1933. doi: 10.1093/brain/awy132, PMID: 29850777 PMC6022632

[ref35] Hayes G. M. Howlett D. R. Griffin G. E. (2002). Production of β-amyloid by primary human Foetal mixed brain cell cultures and its modulation by exogenous soluble β-amyloid. Neuroscience 113, 641–646. doi: 10.1016/S0306-4522(02)00191-4, PMID: 12150783

[ref36] Henley D. Raghavan N. Sperling R. Aisen P. Raman R. Romano G. (2019). Preliminary results of a trial of Atabecestat in preclinical Alzheimer’s disease. N. Engl. J. Med. 380, 1483–1485. doi: 10.1056/NEJMc1813435 30970197

[ref37] Hickman S. Izzy S. Sen P. Morsett L. El Khoury J. (2018). Microglia in neurodegeneration. Nat. Neurosci. 21, 1359–1369. doi: 10.1038/s41593-018-0242-x, PMID: 30258234 PMC6817969

[ref38] Huang X. Cuajungco M. P. Atwood C. S. Hartshorn M. A. Tyndall J. D. A. Hanson G. R.. (1999). Cu(II) potentiation of Alzheimer aβ neurotoxicity. Correlation with cell-free hydrogen peroxide production and metal reduction. J. Biol. Chem. 274, 37111–37116. doi: 10.1074/jbc.274.52.37111, PMID: 10601271

[ref39] Hukins D. Macleod U. Boland J. W. (2019). Identifying potentially inappropriate prescribing in older people with dementia: a systematic review. Eur. J. Clin. Pharmacol. 75, 467–481. doi: 10.1007/s00228-018-02612-x, PMID: 30610274

[ref40] Ittner L. M. Götz J. (2011). Amyloid-β and tau—A toxic pas de deux in Alzheimer’s disease. Nat. Rev. Neurosci. 12, 67–72. doi: 10.1038/nrn2967 21193853

[ref41] Jiang Q. Lee C. Y. D. Mandrekar S. Wilkinson B. Cramer P. Zelcer N.. (2008). ApoE promotes the proteolytic degradation of Aβ. Neuron 58, 681–693. doi: 10.1016/j.neuron.2008.04.010, PMID: 18549781 PMC2493297

[ref42] Jung S. H. Murphy E. A. McClellan J. L. Carmichael M. D. Davis J. M. (2010). The dietary flavonoid quercetin decreases neuroinflammation in a mouse model of Alzheimer’s disease. FASEB J. 24:604.17. doi: 10.1096/FASEBJ.24.1_SUPPLEMENT.604.17

[ref43] Jusko T. A. Henderson C. R. Lanphear B. P. Cory-Slechta D. A. Parsons P. J. Canfield R. L. (2008). Blood lead concentration <10 μg/dL and child intelligence at 6 years of age. Environ. Health Perspect. 116, 243–248. doi: 10.1289/ehp.10424, PMID: 18288325 PMC2235210

[ref44] Kehoe P. G. Miners S. Love S. (2009). Angiotensins in Alzheimer’s disease—friend or foe? Trends Neurosci. 32, 619–628. doi: 10.1016/j.tins.2009.07.006 19796831

[ref45] Kheiri G. Dolatshahi M. Rahmani F. Rezaei N. (2018). Role of p38/MAPKs in Alzheimer’s disease: implications for amyloid beta toxicity targeted therapy. Rev. Neurosci. 30, 9–30. doi: 10.1515/REVNEURO-2018-0008, PMID: 29804103

[ref46] Kihara T. Shimohama S. (2004). Alzheimer’s disease and acetylcholine receptors. Acta Neurobiol. Exp. 64, 99–105. 10.55782/ane-2004-1495 15190684

[ref47] Kim J. Basak J. M. Holtzman D. M. (2009). The role of Apolipoprotein E in Alzheimer’s disease. Neuron 63, 287–303. doi: 10.1016/j.neuron.2009.06.026, PMID: 19679070 PMC3044446

[ref48] Kim E. K. Choi E. J. (2015). Compromised MAPK signaling in human diseases: an update. Arch. Toxicol. 89, 867–882. doi: 10.1007/S00204-015-1472-2, PMID: 25690731

[ref49] Kim H. J. Vaziri N. D. (2010). Contribution of impaired Nrf2-Keap1 pathway to oxidative stress and inflammation in chronic renal failure. Am. J. Physiol. Renal Physiol. 298, F662–F671. doi: 10.1152/ajprenal.00421.2009, PMID: 20007347

[ref50] Kimura T. Whitcomb D. J. Jo J. Regan P. Piers T. Heo S.. (2014). Microtubule-associated protein tau is essential for long-term depression in the hippocampus. Philosoph Transac R Soc B Biol Sci 369:20130144. doi: 10.1098/rstb.2013.0144, PMID: 24298146 PMC3843876

[ref51] Kitazume S. Tachida Y. Kato M. Yamaguchi Y. Honda T. Hashimoto Y.. (2010). Brain endothelial cells produce amyloid β from amyloid precursor protein 770 and preferentially secrete the O-glycosylated form. J. Biol. Chem. 285, 40097–40103. doi: 10.1074/jbc.M110.144626, PMID: 20952385 PMC3000992

[ref52] Kolarova M. García-Sierra F. Bartos A. Ricny J. Ripova D. (2012). Structure and pathology of tau protein in Alzheimer’s disease. Int. J. Alzheimers Dis. 2012, 1–13. doi: 10.1155/2012/731526, PMID: 22690349 PMC3368361

[ref53] Koudinov A. R. Koudinova N. V. (2005). Cholesterol homeostasis failure as a unifying cause of synaptic degeneration. J. Neurol. Sci. 229-230, 233–240. doi: 10.1016/j.jns.2004.11.036, PMID: 15760645

[ref54] Kouroku Y. Fujita E. Tanida I. Ueno T. Isoai A. Kumagai H.. (2007). ER stress (PERK/eIF2α phosphorylation) mediates the polyglutamine-induced LC3 conversion, an essential step for autophagy formation. Cell Death Differ. 14, 230–239. doi: 10.1038/sj.cdd.4401984, PMID: 16794605

[ref55] Lee J. K. Kim N. J. (2017). Recent advances in the inhibition of p38 MAPK as a potential strategy for the treatment of Alzheimer’s disease. Molecules 22, 1–23. doi: 10.3390/molecules22081287, PMID: 28767069 PMC6152076

[ref56] Liao Y. Qi X.-L. Cao Y. Yu W.-F. Ravid R. Winblad B.. (2016). Elevations in the levels of NF-κB and inflammatory chemotactic factors in the brains with Alzheimer’s disease—one mechanism may involve α3 nicotinic acetylcholine receptor. Curr. Alzheimer Res. 13, 1290–1301. doi: 10.2174/1567205013666160703174254, PMID: 27396406

[ref57] Liu C. C. Kanekiyo T. Xu H. Bu G. (2013). Apolipoprotein E and Alzheimer disease: risk, mechanisms and therapy. Nat. Rev. Neurol. 9, 106–118. doi: 10.1038/nrneurol.2012.263, PMID: 23296339 PMC3726719

[ref58] Liu F. Nguyen J. L. Hulleman J. D. Li L. Rochet J. C. (2008). Mechanisms of DJ-1 neuroprotection in a cellular model of Parkinson’s disease. J. Neurochem. 105, 2435–2453. doi: 10.1111/j.1471-4159.2008.05333.x, PMID: 18331584

[ref1] Lovell M. A. Robertson J. D. Teesdale W. J. Campbell J. L. Markesbery W. R. (1998). Copper, iron and zinc in Alzheimer’s disease senile plaques. Int. J. Surg. Pathol. 15, 252–257. doi: 10.1177/1066896907302118 9667777

[ref59] Matsunaga S. Kishi T. Iwata N. (2015). Memantine monotherapy for Alzheimer’s disease:A systematic review and meta-analysis. PLoS One 10, 1–16. doi: 10.1371/journal.pone.0123289, PMID: 25860130 PMC4393306

[ref60] McGleenon B. M. Dynan K. B. Passmore A. P. (2009). Acetylcholinesterase inhibitors and alzheimer’s disease. Encycloped Neurosci 2, 9–13. doi: 10.1016/B978-008045046-9.01129-3 PMC2014378 10583015

[ref61] Mendiola-Precoma J. Berumen L. C. Padilla K. Garcia-Alcocer G. (2016). Therapies for prevention and treatment of Alzheimer’s disease. Biomed. Res. Int. 2016, 1–17. doi: 10.1155/2016/2589276, PMID: 27547756 PMC4980501

[ref62] Miura T. Suzuki K. Kohata N. Takeuchi H. (2000). Metal binding modes of Alzheimer’s amyloid β-peptide in insoluble aggregates and soluble complexes. Biochemistry 39, 7024–7031. doi: 10.1021/bi0002479, PMID: 10841784

[ref63] Mordn M. A. Mufson E. J. G6mez-Ramos P. Morcillo A. (1993). Acta Heuropathologlca regular papers Colocalization of cholinesterases with 5 amyloid protein in aged and Alzheimer’s brains*. Acta Neuropathol. 85, 362–369. doi: 10.1007/BF00334445 8480510

[ref64] Mount C. Downton C. (2006). Alzheimer: progress o profit. Nat. Med. 12, 780–784. doi: 10.1038/nm0706-780 16829947

[ref65] Nair S. Doh S. T. Chan J. Y. Kong A. N. Cai L. (2008). Regulatory potential for concerted modulation of Nrf2- and Nfkb1-mediated gene expression in inflammation and carcinogenesis. Br. J. Cancer 99, 2070–2082. doi: 10.1038/SJ.BJC.6604703, PMID: 19050705 PMC2607222

[ref66] Nelson L. Tabet N. (2015). Slowing the progression of Alzheimer’s disease; what works? Ageing Res. Review. 23, 193–209. doi: 10.1016/j.arr.2015.07.002 26219494

[ref67] Neurology T. L. (2016). Solanezumab: too late in mild Alzheimer’ s disease? Lancet Neurol. 16:97. doi: 10.1016/S1474-4422(16)30395-7, PMID: 28102152

[ref68] Nichols E. Szoeke C. E. I. Vollset S. E. Abbasi N. Abd-Allah F. Abdela J.. (2019). Global, regional, and national burden of Alzheimer’s disease and other dementias, 1990–2016: a systematic analysis for the global burden of disease study 2016. Lancet Neurol. 18, 88–106. doi: 10.1016/S1474-4422(18)30403-4, PMID: 30497964 PMC6291454

[ref69] O’brien R. J. Wong P. C. (2011). Amyloid precursor protein processing and Alzheimer’s disease. Annu Rev Sci. 34, 185–204. doi: 10.1146/annurev-neuro-061010-113613, PMID: 21456963 PMC3174086

[ref70] Ogata M. Hino S. Saito A. Morikawa K. Kondo S. Kanemoto S.. (2006). Autophagy is activated for cell survival after endoplasmic reticulum stress. Mol. Cell. Biol. 26, 9220–9231. doi: 10.1128/mcb.01453-06, PMID: 17030611 PMC1698520

[ref71] Olajide O. A. Sarker S. D. (2020). Alzheimer’s disease: natural products as inhibitors of neuroinflammation. Inflammopharmacology 28, 1439–1455. doi: 10.1007/s10787-020-00751-1, PMID: 32930914 PMC7572326

[ref72] Osenkowski P. Ye W. Wang R. Wolfe M. S. Selkoe D. J. (2008). Direct and potent regulation of γ-secretase by its lipid microenvironment. J. Biol. Chem. 283, 22529–22540. doi: 10.1074/jbc.M801925200, PMID: 18539594 PMC2504869

[ref73] Parent M. J. Bedard M.-A. Aliaga A. Minuzzi L. Mechawar N. Soucy J.-P.. (2013). Cholinergic depletion in Alzheimer’s disease shown by [18F]FEOBV autoradiography. Int J Mol Imaging 2013, 1–6. doi: 10.1155/2013/205045, PMID: 24324884 PMC3844185

[ref74] Prasansuklab A. Tencomnao T. (2013). Amyloidosis in Alzheimer’s disease: the toxicity of amyloid Beta (Aβ), mechanisms of its accumulation and implications of medicinal plants for therapy. Evid. Based Complement. Alternat. Med. 2013, 1–10. doi: 10.1155/2013/413808, PMID: 23762130 PMC3671299

[ref75] Prentice H. Modi J. P. Wu J. Y. (2015). Mechanisms of neuronal protection against Excitotoxicity, endoplasmic reticulum stress, and mitochondrial dysfunction in stroke and neurodegenerative diseases. Oxid. Med. Cell. Longev. 2015, 1–7. doi: 10.1155/2015/964518, PMID: 26576229 PMC4630664

[ref76] Prince M. Albanese E. Guerchet M. Prina M. (2014). World Alzheimer report 2014: Dementia and risk reduction. An Analysis of Protective and Modifiable Factors. Alzheimers Disease International. London.

[ref77] Qi X. L. Xiu J. Shan K. R. Xiao Y. Gu R. Liu R. Y.. (2005). Oxidative stress induced by beta-amyloid peptide1-42 is involved in the altered composition of cellular membrane lipids and the decreased expression of nicotinic receptors in human SH-SY5Y neuroblastoma cells. Neurochem. Int. 46, 613–621. doi: 10.1016/j.neuint.2005.02.007, PMID: 15863239

[ref78] Quiroz-Baez R. Rojas E. Arias C. (2009). Oxidative stress promotes JNK-dependent amyloidogenic processing of normally expressed human APP by differential modification of α-, β- and γ-secretase expression. Neurochem. Int. 55, 662–670. doi: 10.1016/j.neuint.2009.06.012, PMID: 19560504

[ref79] Ramsey C. P. Glass C. A. Montgomery M. B. Lindl K. A. Ritson G. P. Chia L. A.. (2007). Expression of Nrf2 in neurodegenerative diseases. J. Neuropathol. Exp. Neurol. 66, 75–85. doi: 10.1097/nen.0b013e31802d6da9, PMID: 17204939 PMC2253896

[ref80] Rankin C. A. Sun Q. Gamblin T. C. (2007). Tau phosphorylation by GSK-3ß promotes tangle-like filament morphology. Mol. Neurodegener. 2, 1–14. doi: 10.1186/1750-1326-2-12, PMID: 17598919 PMC1936422

[ref81] Reitz C. (2012). Alzheimer’s disease and the amyloid cascade hypothesis: A critical review. Int. J. Alzheimers Dis. 2012:369808. doi: 10.1155/2012/369808, PMID: 22506132 PMC3313573

[ref82] Religa D. Strozyk D. Cherny R. A. Volitakis I. Haroutunian V. Winblad B.. (2006). Elevated cortical zinc in Alzheimer disease. Neurology 67, 69–75. doi: 10.1212/01.wnl.0000223644.08653.b5 16832080

[ref83] Riyaz Basha M. Wei W. Bakheet S. A. Benitez N. Siddiqi H. K. Ge Y.-W.. (2005). The fetal basis of Amyloidogenesis: exposure to Lead and latent overexpression of amyloid precursor protein and-amyloid in the aging brain. J. Neurosci. 25, 823–829. doi: 10.1523/JNEUROSCI.4335-04.2005, PMID: 15673661 PMC6725614

[ref84] Rojo A. I. Innamorato N. G. Martín-Moreno A. M. De Ceballos M. L. Yamamoto M. Cuadrado A. (2010). Nrf2 regulates microglial dynamics and neuroinflammation in experimental Parkinson’s disease. Glia 58, 588–598. doi: 10.1002/GLIA.20947, PMID: 19908287

[ref85] Sagare A. Deane R. Bell R. D. Johnson B. Hamm K. Pendu R.. (2007). Clearance of amyloid-β by circulating lipoprotein receptors. Nat. Med. 13, 1029–1031. doi: 10.1038/nm1635, PMID: 17694066 PMC2936449

[ref86] Sandberg M. Patil J. D’Angelo B. Weber A S. G. Mallard C. (2014). NRF2-regulation in brain health and disease: implication of cerebral inflammation. Neuropharmacology 79, 298–306. doi: 10.1016/j.neuropharm.2013.11.004.NRF2-regulation, PMID: 24262633 PMC3958930

[ref87] Sberna G. Sáez-Valero J. Beyreuther K. Masters C. L. Small D. H. (1997). The amyloid β-protein of Alzheimer’s disease increases acetylcholinesterase expression by increasing intracellular calcium in embryonal carcinoma P19 cells. J. Neurochem. 69, 1177–1184. doi: 10.1046/j.1471-4159.1997.69031177.x, PMID: 9282941

[ref88] Schmitt T. L. Steiner E. Klingler P. Lassmann H. Grubeck-Loebenstein B. (1995). Thyroid epithelial cells produce large amounts of the Alzheimer β-amyloid precursor protein (APP) and generate potentially amyloidogenic APP fragments. J. Clin. Endocrinol. Metab. 8, 3513–3519. 10.1210/jcem.80.12.8530592 8530592

[ref89] Schnöder L. Hao W. Qin Y. Liu S. Tomic I. Liu X.. (2016). Deficiency of neuronal p38α MAPK attenuates amyloid pathology in Alzheimer disease mouse and cell models through facilitating lysosomal degradation of BACE1. J. Biol. Chem. 291, 2067–2079. doi: 10.1074/jbc.M115.695916, PMID: 26663083 PMC4732195

[ref90] Selkoe D. J. (2001). Clearing the brain’s amyloid cobwebs. Neuron 32, 177–180. doi: 10.1016/S0896-6273(01)00475-5, PMID: 11683988

[ref91] Storey E. Cappai R. (1999). The amyloid precursor protein of Alzheimer’s disease and the Aβ peptide. Neuropathol. Appl. Neurobiol. 25, 81–97. doi: 10.1046/j.1365-2990.1999.00164.x 10215996

[ref92] Suganthy N. Devi K. P. Nabavi S. F. Braidy N. Nabavi S. M. (2016). Bioactive effects of quercetin in the central nervous system: focusing on the mechanisms of actions. Biomed. Pharmacother. 84, 892–908. doi: 10.1016/j.biopha.2016.10.011, PMID: 27756054

[ref93] Thinakaran G. Koo E. H. (2008). Amyloid precursor protein trafficking, processing, and function. J. Biol. Chem. 283, 29615–29619. doi: 10.1074/JBC.R800019200, PMID: 18650430 PMC2573065

[ref94] Tong Y. Zhou W. Fung V. Christensen M. A. Qing H. Sun X.. (2005). Oxidative stress potentiates BACE1 gene expression and Aβ generation. J. Neural Transm. 112, 455–469. doi: 10.1007/s00702-004-0255-3, PMID: 15614428

[ref95] Van Dyck C. H. (2018). Anti-amyloid-β monoclonal antibodies for Alzheimer’s disease: pitfalls and promise. Physiol. Behav. 83, 311–319. doi: 10.1016/j.biopsych.2017.08.010.Anti-Amyloid-, PMID: 28967385 PMC5767539

[ref96] Vandenberghe R. Rinne J. O. Boada M. Katayama S. Scheltens P. Vellas B.. (2016). Bapineuzumab for mild to moderate Alzheimer’s disease in two global, randomized, phase 3 trials. Alzheimers Res. Ther. 8:18. doi: 10.1186/s13195-016-0189-7, PMID: 27176461 PMC4866415

[ref97] Vinod T. Kuhad A. Bishnoi M. Chopra K. (2009). Chronic treatment with tocotrienol, an isoform of vitamin E, prevents intracerebroventricular streptozotocin-induced cognitive impairment and oxidative-nitrosative stress in rats. Pharmacol. Biochem. Behav. 93, 183–189. doi: 10.1016/j.pbb.2009.05.009, PMID: 19464315

[ref98] Wimo A. Elenn Guerchet M. Ali G.-C. Wu Y.-T. Prina A. M. Winblad B.. (2017). The worldwide costs of dementia 2015 and comparisons with 2010. Alzheimers Dement. 13, 1–7. doi: 10.1016/j.jalz.2016.07.150, PMID: 27583652 PMC5232417

[ref99] Winston W. (2020). Economic burden of Alzheimer disease and managed care considerations. Am. J. Manag. Care 26, S177–S183. doi: 10.37765/AJMC.2020.88482, PMID: 32840331

[ref100] Yiannopoulou K. G. Papageorgiou S. G. (2013). Current and future treatments for Alzheimer’s disease. Ther. Adv. Neurol. Disord. 6, 19–33. doi: 10.1177/1756285612461679, PMID: 23277790 PMC3526946

[ref101] Yiannopoulou K. G. Papageorgiou S. (2020). Current and future treatments in Alzheimer disease: an update. J. Central Nervous Syst Dis 12, 117957352090739–117957352090712. doi: 10.1177/1179573520907397, PMID: 32165850 PMC7050025

[ref102] Yin J. C. P. Wallach J. S. Del Vecchio M. Wilder E. L. Zhou H. Quinn W. G.. (1994). Induction of a dominant negative CREB transgene specifically blocks long-term memory in drosophila. Cells 79, 49–58. doi: 10.1016/0092-8674(94)90399-9, PMID: 7923376

[ref103] Youssef E.-H. H. Wiley R. E. Khoury C. P. Daya R. P. Ballard C. Evans A. R.. (2019). Tip of the iceberg: assessing the global socioeconomic costs of Alzheimer’s disease and related dementias and strategic implications for stakeholders. J. Alzheimers Dis. 70, 323–341. doi: 10.3233/JAD-190426, PMID: 31256142 PMC6700654

[ref104] Yu M. Li H. Liu Q. Liu F. Tang L. Li C.. (2011). Nuclear factor p65 interacts with Keap1 to repress the Nrf2-ARE pathway. Cell. Signal. 23, 883–892. doi: 10.1016/J.CELLSIG.2011.01.014, PMID: 21262351

[ref105] Zhang X. Fu Z. Meng L. He M. Zhang Z. (2018). The early events that initiate β-amyloid aggregation in Alzheimer’s disease. Front. Aging Neurosci. 10, 1–13. doi: 10.3389/fnagi.2018.00359, PMID: 30542277 PMC6277872

[ref106] Zhou F. Du G. Xie J. Gu J. Jia Q. Fan Y.. (2020). RyRs mediate lead-induced neurodegenerative disorders through calcium signaling pathways. Sci. Total Environ. 701:134901. doi: 10.1016/j.scitotenv.2019.134901, PMID: 31710906

[ref107] Zieg J. Greer P. L. Greenberg M. E. (2008). SnapShot: Ca2+-dependent transcription in neurons. Cells 134, 1080–1080.e2. doi: 10.1016/j.cell.2008.09.010, PMID: 18805099

